# Supplementary material for: Genomic Footprints of Selfing, Introduction History, and Long-Distance Dispersal in an Invasive Alien Plant
Source: Genome Biol Evol. 2026 May 22;18(6):evag124. doi: 10.1093/gbe/evag124 (PMC13228144; doi:10.1093/gbe/evag124)
Supplement: evag124_Supplementary_Data [file evag124_supplementary_data.docx]

**Genomic footprints of selfing, introduction history, and long-distance dispersal in a highly invasive alien plant**

**SUPPLEMENTARY MATERIAL**

Raúl Sánchez-García^1*^, Andy J. Green^1,2^, María A. Ortiz^3^, Cristina García^4^, Francisco Hortas^5^, Chevonne Reynolds^6^, Jennifer Rowntree^7^, Ester A. Serrão^8^, Lina Tomasson^9^, Karin Tremetsberger^10^, Casper H.A. van Leeuwen^11^, and Joaquín Ortego^12^

^1^ Departamento de Biología de la Conservación y Cambio Global, Estación Biológica de Doñana (EBD), CSIC, Seville, Spain

^2^ Department of Natural Sciences, Manchester Metropolitan University, Manchester, UK

^3^ Department of Plant Biology and Ecology, University of Sevilla, Seville, Spain

^4^ Department of Biological Sciences, School of Life and Environmental Sciences, Royal Holloway University of London, Egham, Surrey, UK

^5^ Department of Biology, Institute of Marine Research (INMAR), University of Cadiz, Puerto Real, Spain

^6^ School of Animal, Plant and Environmental Sciences, University of the Witwatersrand, Johannesburg, South Africa

^7^ School of Biological and Marine Sciences, University of Plymouth, Drake Circus, Plymouth, UK

^8^ Centre of Marine Sciences, CCMAR, University of Algarve, Campus de Gambelas, Faro, Portugal

^9^ National Coordinator for Aquatic Invasive Alien Species, Swedish Agency for Marine and Water Management, Gothenburg, Sweden

^10^ BOKU University, Institute of Botany, Department of Ecosystem Management, Climate and Biodiversity, Vienna, Austria

^11^ Department of Ecology, Radboud Institute for Biological and Environmental Sciences, Radboud University, Nijmegen, the Netherlands

^12^ Departamento de Ecología y Evolución, Estación Biológica de Doñana (EBD), CSIC, Seville, Spain

**
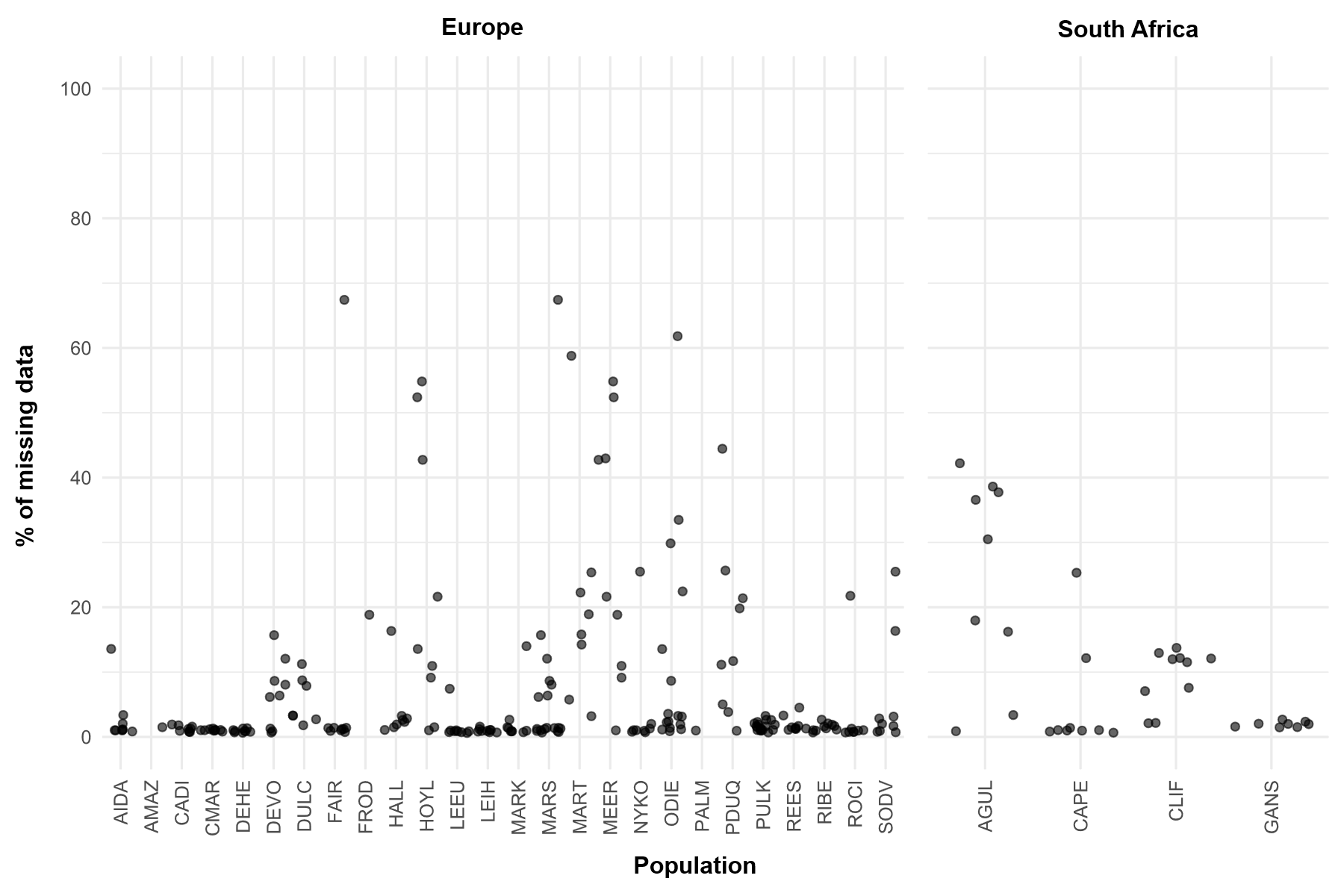
**

Fig. S1. Distribution of missing data per individual for the dataset of *Cotula coronopifolia* including all 266 individuals (6,661 unlinked SNPs). The y-axis represents the percentage of missing data derived from ipyrad SNP calling, and the x-axis shows the sampled populations grouped by geographic region: 26 European populations (left panel) and four South African populations (right panel).


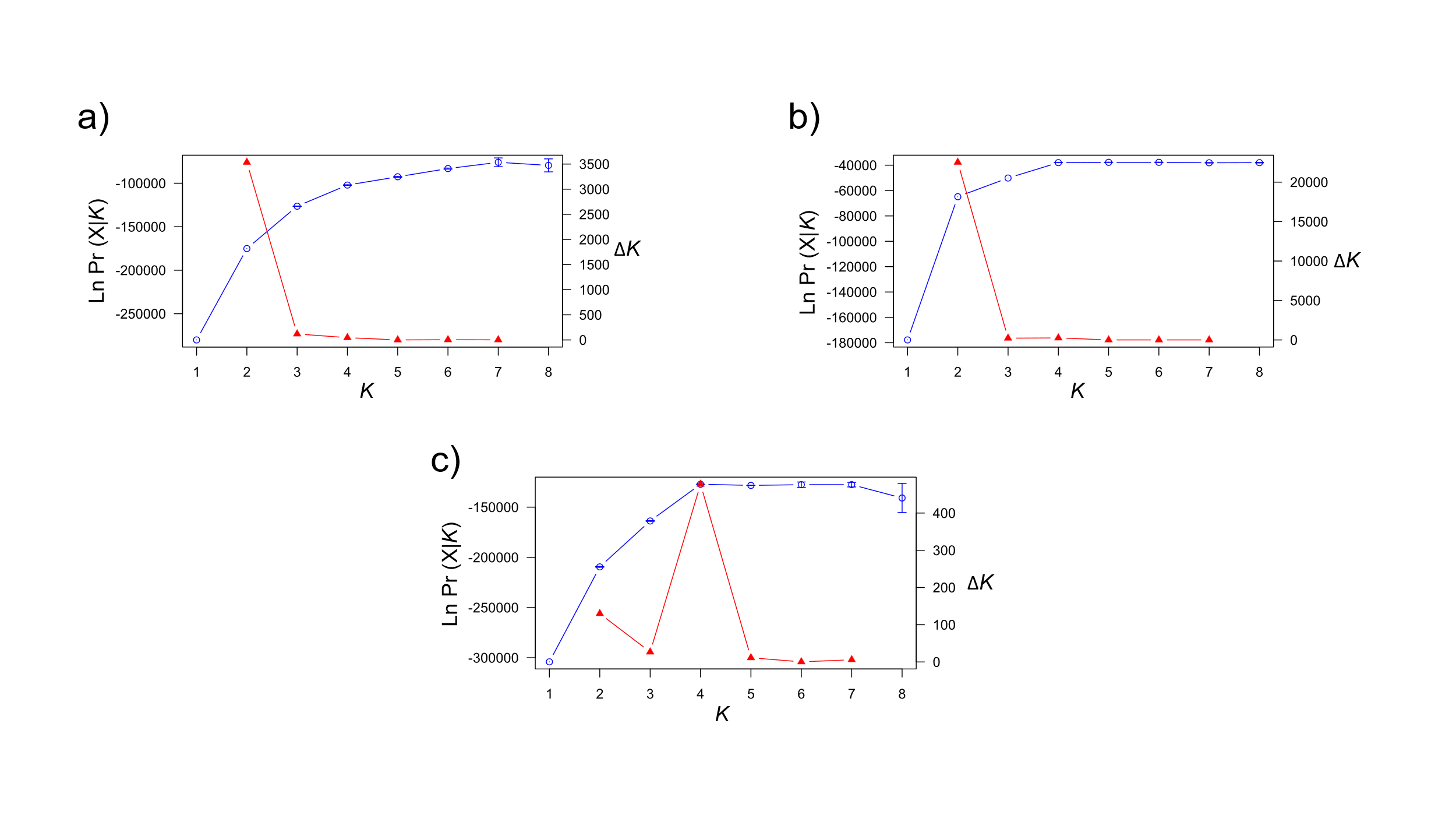


Fig. S2. Mean (±SD) log probability of the data (LnPr(X|*K*)) over 10 runs of structure (left axes, blue dots and error bars) for each value of *K* and the corresponding magnitude of Δ*K* (right axes, red triangles) for datasets of *Cotula coronopifolia* (a) including all 266 individuals, (b) 230 individuals from Europe, and (c) 36 individuals from South Africa.


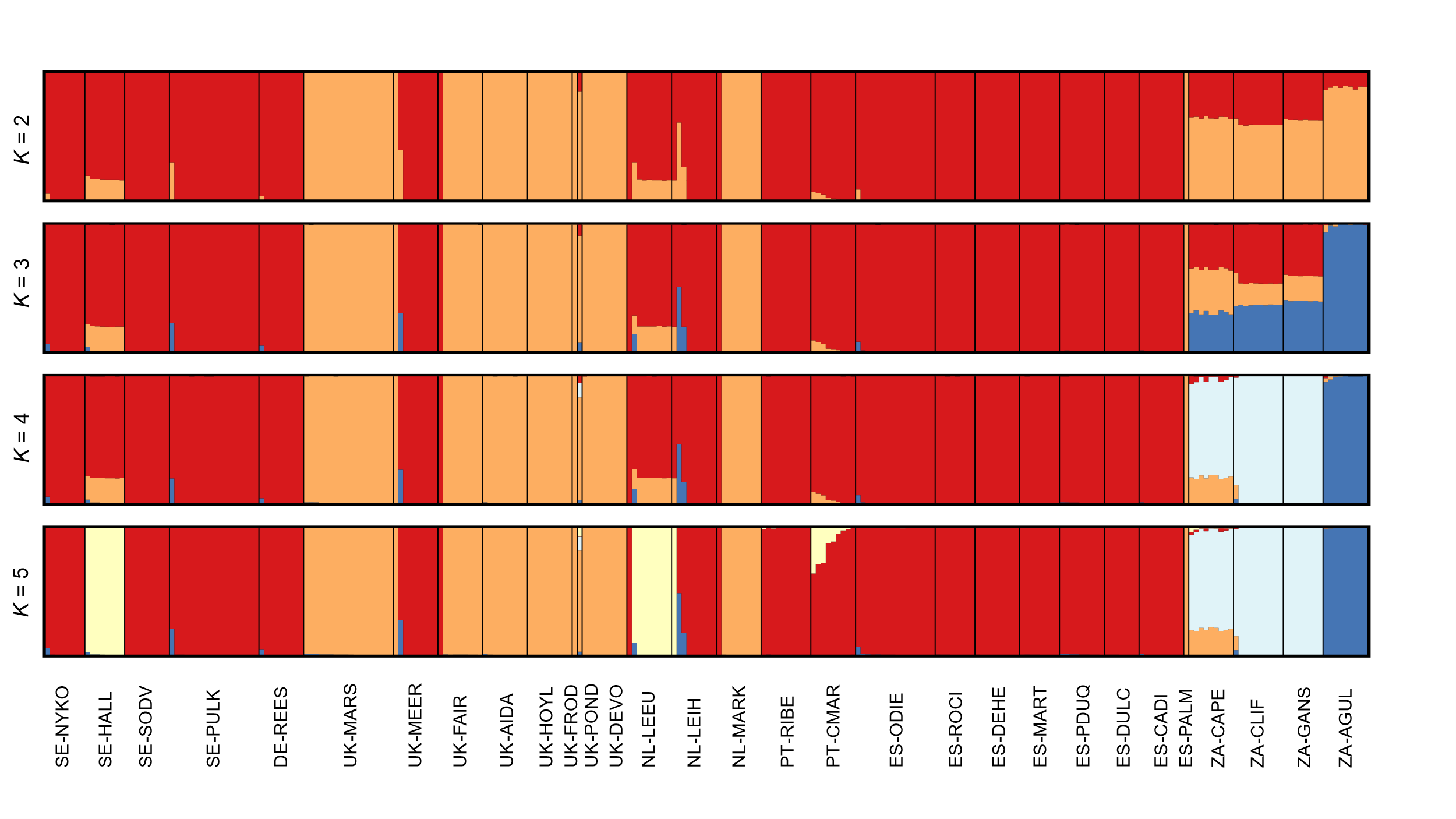


Fig. S3. Results of genetic assignments based on the Bayesian clustering analyses implemented in structure (*K* = 2-5) for the dataset of *Cotula coronopifolia* including all 266 individuals (6,661 SNPs). Each individual is represented by a vertical bar, which is partitioned into *K* coloured segments showing the individual’s probability of belonging to the cluster with that colour. Thin vertical black lines separate individuals from different populations. Population codes as described in Table 1.


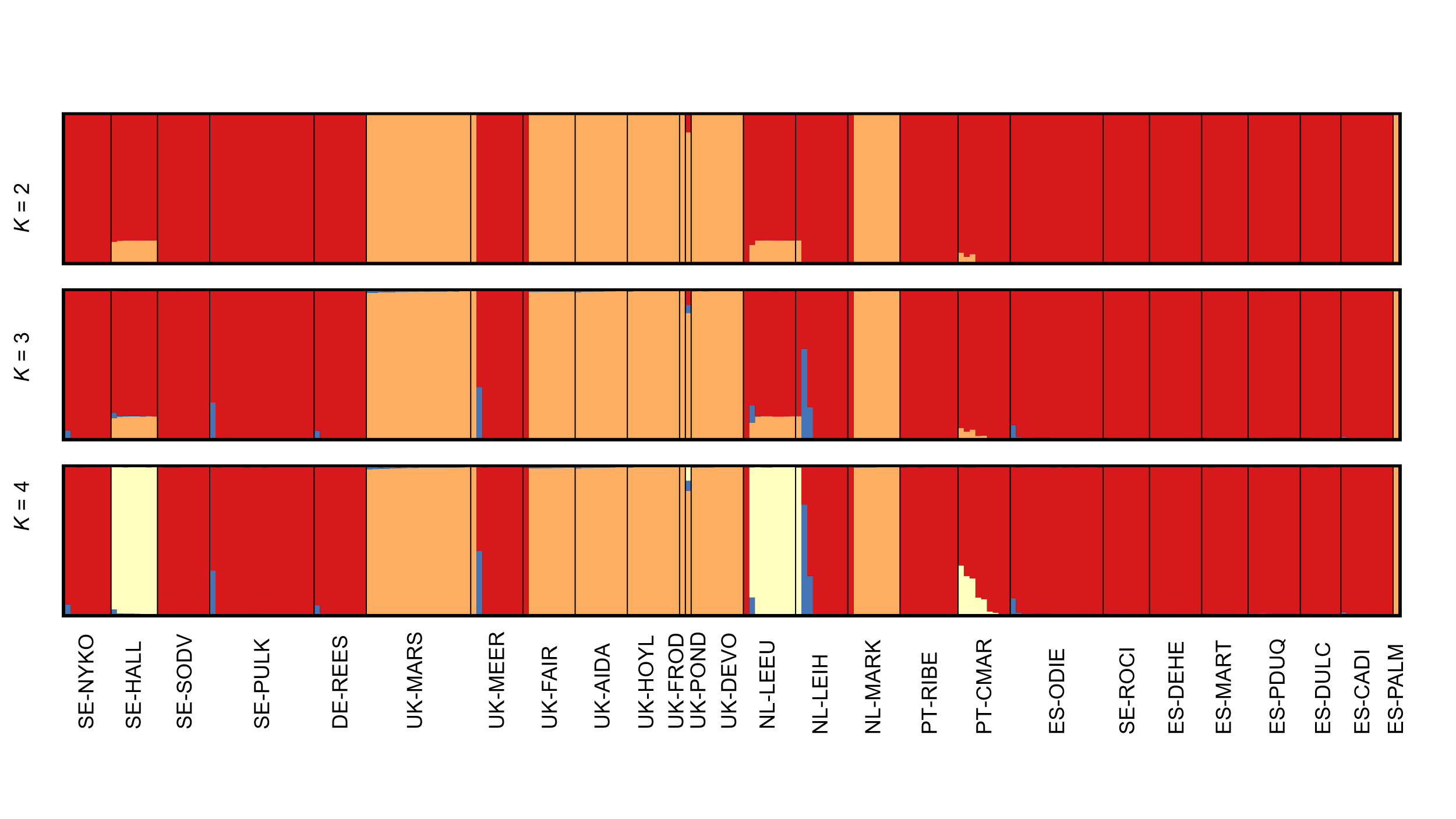


Fig. S4. Results of genetic assignments based on the Bayesian clustering analyses implemented in the program structure (*K* = 2-4) for the dataset of *Cotula coronopifolia* including 230 individuals from Europe (5,692 SNPs). Each individual is represented by a vertical bar, which is partitioned into *K* coloured segments showing the individual’s probability of belonging to the cluster with that colour. Thin vertical black lines separate individuals from different populations. Population codes as described in Table 1.


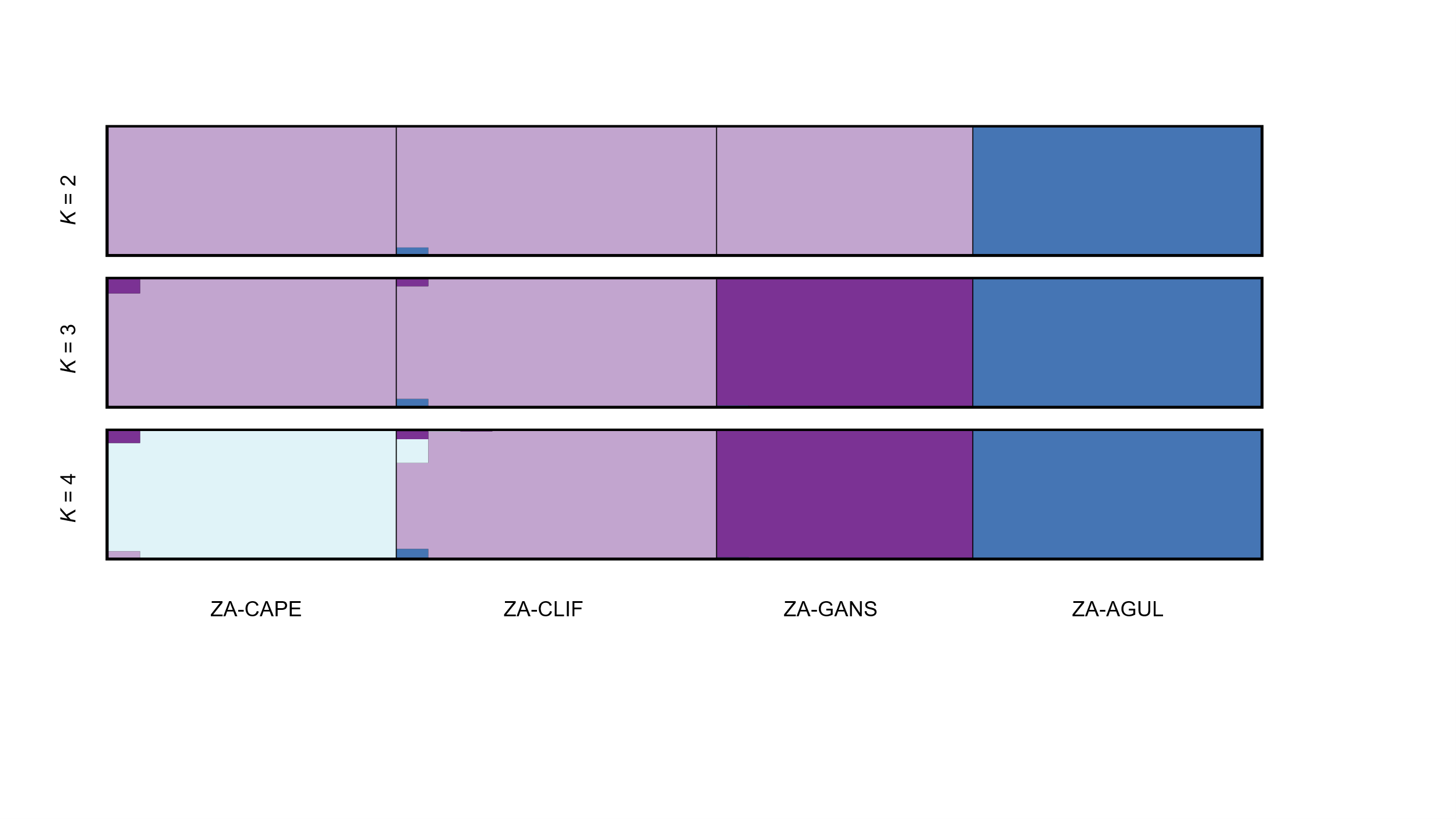


Fig. S5. Results of genetic assignments based on the Bayesian clustering analyses implemented in the program structure (*K* = 2-4) for the dataset of *Cotula coronopifolia* including 36 individuals from South Africa (13,079 SNPs). Each individual is represented by a vertical bar, which is partitioned into *K* coloured segments showing the individual’s probability of belonging to the cluster with that colour. Thin vertical black lines separate individuals from different populations. Population codes as described in Table 1.


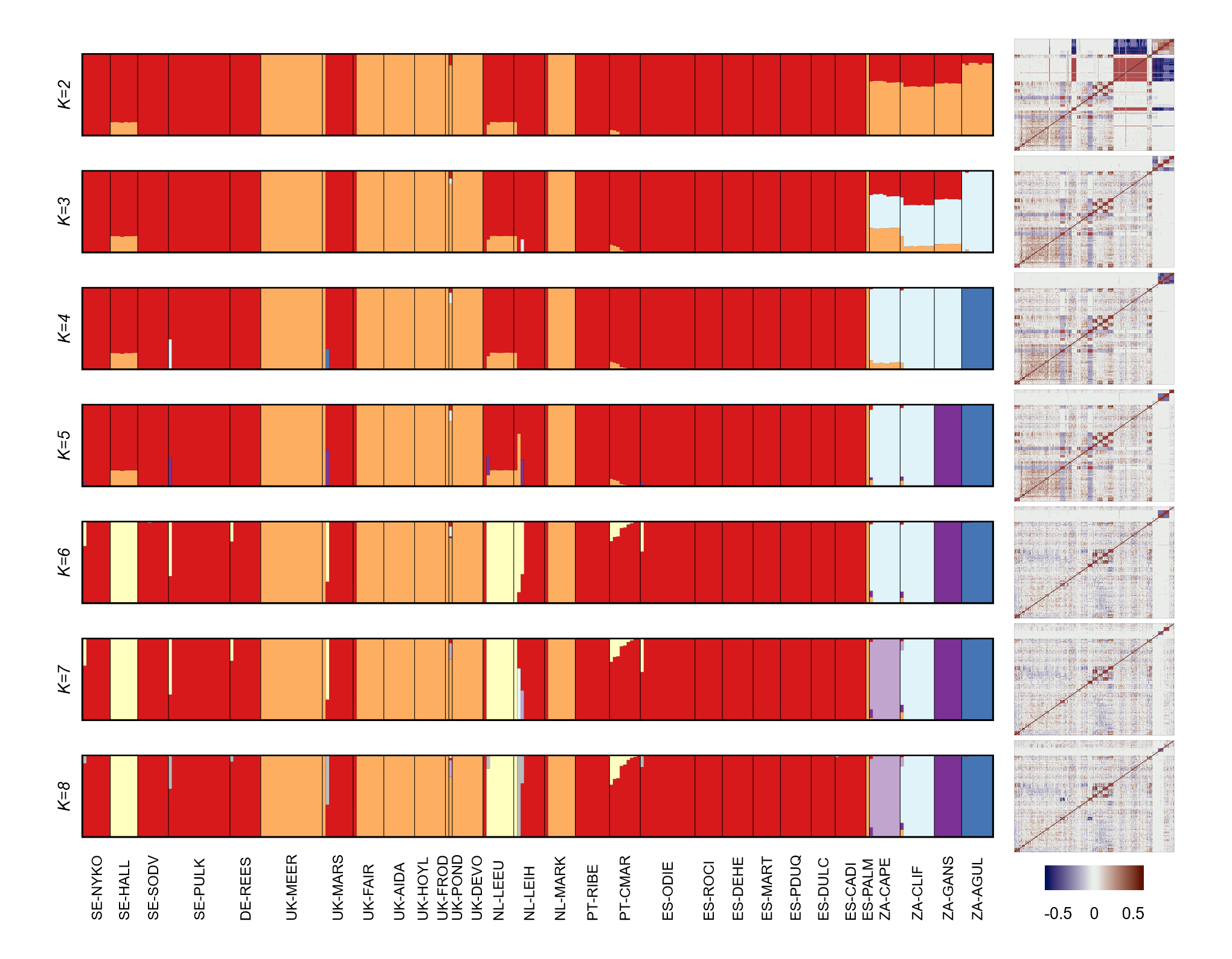


Fig. S6. Results of genetic assignments based on the maximum-likelihood approach implemented in admixture (*K* = 2-8) for the dataset of *Cotula coronopifolia* including all 266 individuals (6,661 SNPs). Left panel: Bar plots showing individual ancestry proportions. Each individual is represented by a vertical bar, which is partitioned into *K* coloured segments showing the individual’s probability of belonging to the cluster with that colour. Thin vertical black lines separate individuals from different populations. Population codes as described in Table 1. Right panel: Heatmaps display the correlation of residuals between individuals for each value of *K*. Positive (red) and negative (blue) correlations indicate a poor fit to the model, while values near zero (white) indicate a robust fit.


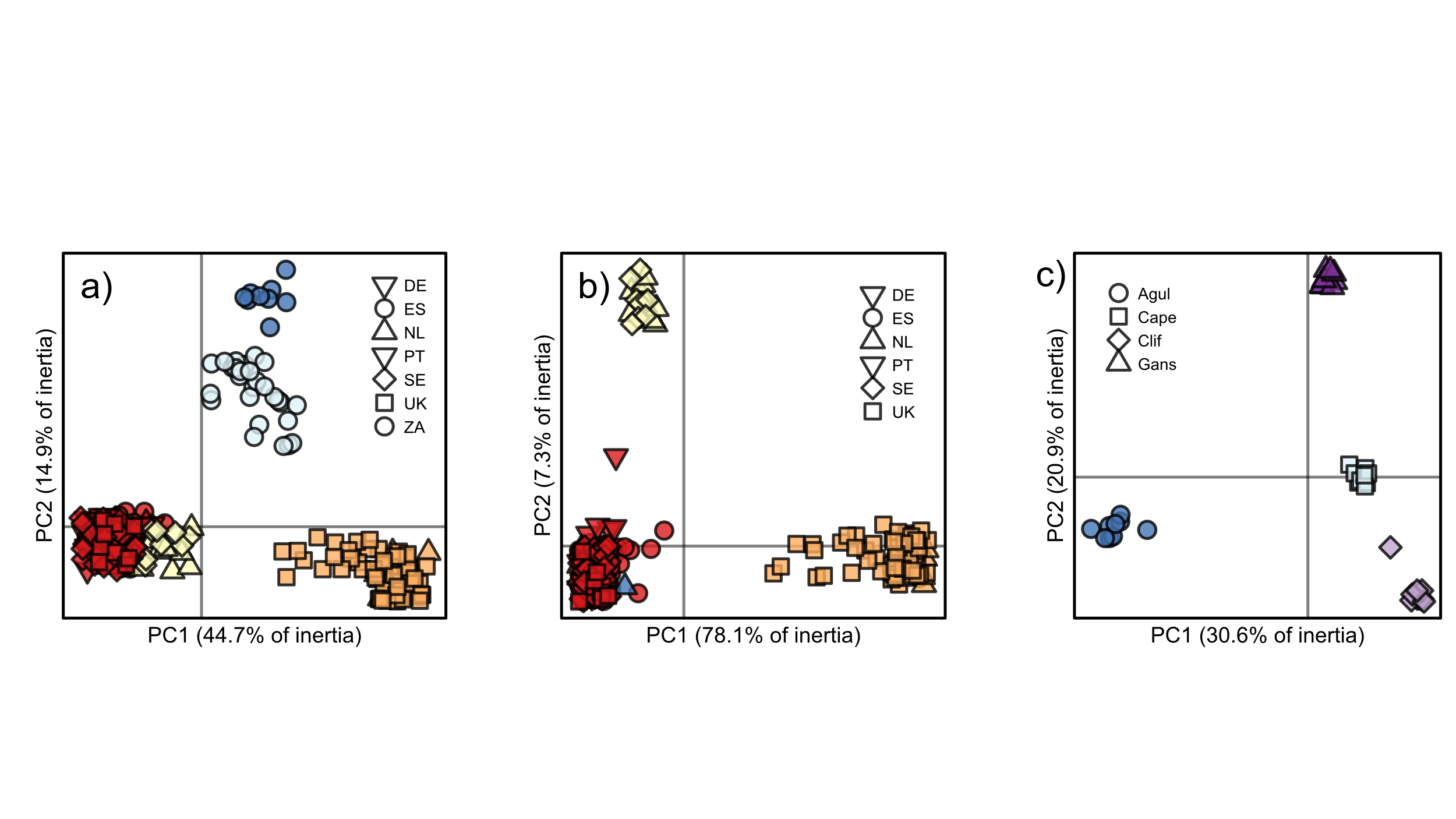


Fig. S7. Principal Component Analyses (PCA) of genetic variation for datasets of *Cotula coronopifolia* (a) including all 266 individuals (6,661 SNPs), (b) 230 individuals from Europe (5,692 SNPs), and (c) 36 individuals from South Africa (13,079 SNPs). Points are coloured according to the cluster with the highest assignment probability inferred from structure analyses for each dataset. Point shapes indicate the country of origin.


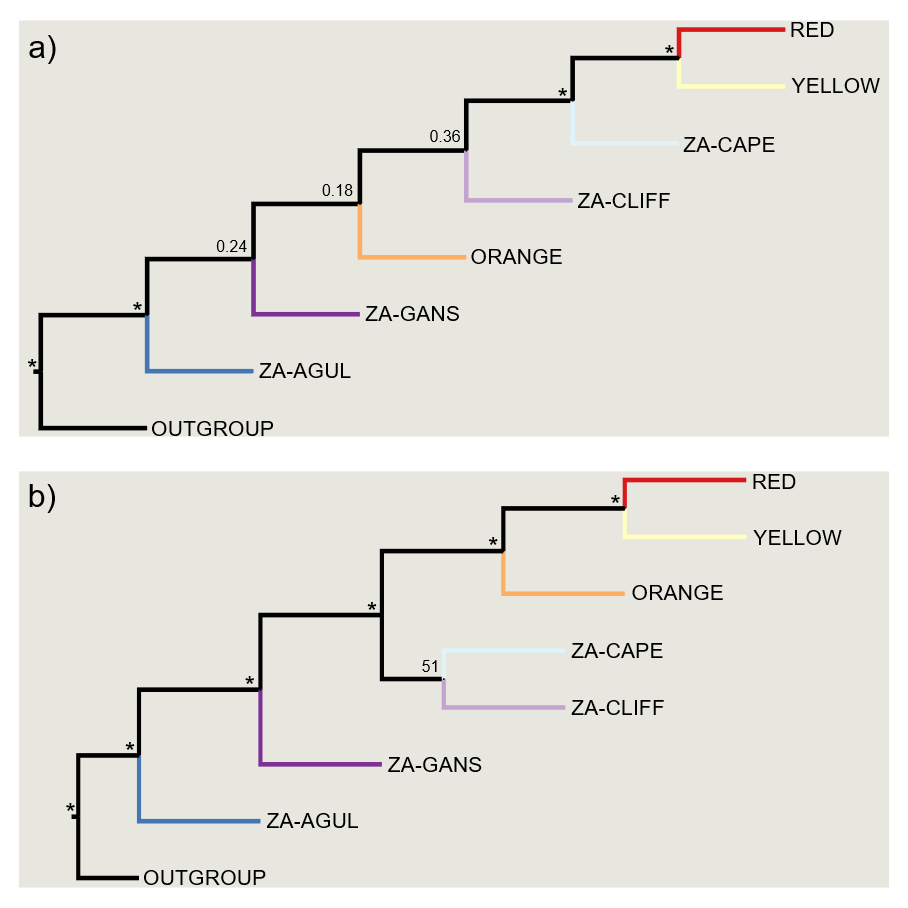


Fig. S8. Phylogenetic trees inferred for lineages of *Cotula coronopifolia* using (a) bpp (analyses A01) and (b) svdquartets. Numbers at nodes indicate (a) posterior probability (PP) support values (* = 1) and (b) bootstrap support values (* = 100).


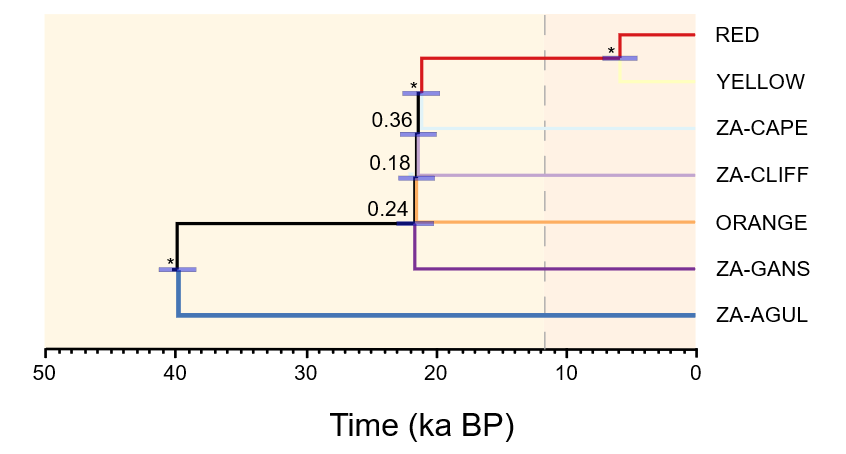


Fig. S9. Phylogenetic tree inferred with bpp (analysis A01) and divergence times estimated using bpp (analysis A00) for lineages of *C. coronopifolia* identified by the Bayesian clustering analyses implemented in structure in the European invasive range (lineages RED, ORANGE and YELLOW) and the South African native range (populations ZA-CAPE, ZA-CLIFF, ZA-GANS, and ZA-AGUL). Bars on nodes indicate 95% highest posterior densities (HPD) intervals of divergence times estimated considering a genomic mutation rate of 7.0 × 10^−9^ substitutions per site per generation and a one-year generation time; numbers at nodes indicate posterior probability (PP) support values (* = 1). Branch colours correspond to the genetic clusters inferred by structure (see Fig. 2). Background shading indicates geological divisions of the Quaternary, and the vertical dashed line marks the boundary between the Late Pleistocene (left) and the Holocene (right).
